# 

**Published:** 1856-02

**Authors:** 


					﻿TO CORRESPONDENTS.
■Æ	*° tte Edi‘,>r“‘1 »f «“
At	C?“m“nica*ions> having reference to the Subscription, the Advertising
e Financial Department, should be addressed to the Treasurer J G West
mobeland, M. D., Dean of the Faculty of the Atlanta Medical College. ’
ft®“ Messrs. A. Tilden and W. II. Phillpot are authorized Agents for this
Journal.
PRIVATE MEDICAL INSTRUCTION.
design t0 give a Course of Private Instruction to such Medi-
cal 8tudents as may desire to enter their office. Regular lectures and examinations
on the various branches of medical science will be afforded twice a week
Atlant. h™* i 10RR	X G* WESTMORELAND, M. D.,
Atlanta, Sept. 1, 1855.	W. F. WESTMORELAND, M. D.
				

